# Safety and Tolerability of CSL112, a Reconstituted, Infusible, Plasma-Derived Apolipoprotein A-I, After Acute Myocardial Infarction: The AEGIS-I Trial (ApoA-I Event Reducing in Ischemic Syndromes I)

**DOI:** 10.1161/CIRCULATIONAHA.116.025687

**Published:** 2016-12-12

**Authors:** C. Michael Gibson, Serge Korjian, Pierluigi Tricoci, Yazan Daaboul, Megan Yee, Purva Jain, John H. Alexander, P. Gabriel Steg, A. Michael Lincoff, John J.P. Kastelein, Roxana Mehran, Denise M. D’Andrea, Lawrence I. Deckelbaum, Bela Merkely, Maciej Zarebinski, Ton Oude Ophuis, Robert A. Harrington

**Affiliations:** From PERFUSE Study Group, Cardiovascular Division, Department of Medicine, Beth Israel Deaconess Medical, Harvard Medical School, Boston, MA (C.M.G., S.K., Y.D., M.Y., P.J.); Duke Clinical Research Institute, Cardiovascular Division, Department of Medicine, Duke University Health, Durham, NC (P.T., J.H.A.); INSERM-Unité 1148, France Assistance Publique-Hôpitaux de Paris, Hôpital Bichat, France Université Paris-Diderot, Sorbonne- Paris Cité, France National Heart and Lung Institute, Paris, France (P.G.S.); Imperial College London, UK Institute of Cardiovascular Medicine and Science, and Royal Brompton Hospital, London, UK (P.G.S.); Department of Cardiovascular Medicine, Cleveland Clinic Foundation, Cleveland, OH (A.M.L.); Department of Vascular Medicine, Academic Medical Center, University of Amsterdam, Amsterdam, the Netherlands (J.J.P.K.); Cardiovascular Institute, Mount Sinai Medical Center, Icahn School of Medicine at Mount Sinai, New York (R.M.); CSL Behring, LLC, King of Prussia, PA (D.M.D., L.I.D.); Heart and Vascular Center, Semmelweis University, Budapest, Hungary (B.M.); Department of Cardiology, Warsaw Medical University, Warsaw, Poland (M.Z.); Department of Cardiology, Canisius Wilhelmina Ziekenhuis, Nijmegen, the Netherlands (T.O.O.); and Department of Medicine, Stanford University, Stanford, CA (R.A.H.).

**Keywords:** alipoprotein A-I, myocardial infarction

## Abstract

**Background:**

Human or recombinant apolipoprotein A-I (apoA-I) has been shown to increase high-density lipoprotein–mediated cholesterol efflux capacity and to regress atherosclerotic disease in animal and clinical studies. CSL112 is an infusible, plasma-derived apoA-I that has been studied in normal subjects or those with stable coronary artery disease. This study aimed to characterize the safety, tolerability, pharmacokinetics, and pharmacodynamics of CSL112 in patients with a recent acute myocardial infarction.

**Methods:**

The AEGIS-I trial (Apo-I Event Reducing in Ischemic Syndromes I) was a multicenter, randomized, double-blind, placebo-controlled, dose-ranging phase 2b trial. Patients with myocardial infarction were stratified by renal function and randomized 1:1:1 to CSL112 (2 g apoA-I per dose) and high-dose CSL112 (6 g apoA-I per dose), or placebo for 4 consecutive weekly infusions. Coprimary safety end points were occurrence of either a hepatic safety event (an increase in alanine transaminase >3 times the upper limit of normal or an increase in total bilirubin >2 times the upper limit of normal) or a renal safety event (an increase in serum creatinine >1.5 times the baseline value or a new requirement for renal replacement therapy).

**Results:**

A total of 1258 patients were randomized, and 91.2% received all 4 infusions. The difference in incidence rates for an increase in alanine transaminase or total bilirubin between both CSL112 arms and placebo was within the protocol-defined noninferiority margin of 4%. Similarly, the difference in incidence rates for an increase in serum creatinine or a new requirement for renal replacement therapy was within the protocol-defined noninferiority margin of 5%. CSL112 was associated with increases in apoA-I and ex vivo cholesterol efflux similar to that achieved in patients with stable coronary artery disease. In regard to the secondary efficacy end point, the risk for the composite of major adverse cardiovascular events among the groups was similar.

**Conclusions:**

Among patients with acute myocardial infarction, 4 weekly infusions of CSL112 are feasible, well tolerated, and not associated with any significant alterations in liver or kidney function or other safety concern. The ability of CSL112 to acutely enhance cholesterol efflux was confirmed. The potential benefit of CSL112 to reduce major adverse cardiovascular events needs to be assessed in an adequately powered phase 3 trial.

**Clinical Trial Registration:**

URL: https://clinicaltrials.gov. Unique identifier: NCT02108262.

## Abstract 2

Supplemental Digital Content is available in the text.

Despite advances in therapeutic strategies for acute myocardial infarction (MI), patients remain at a high risk for recurrent ischemic events, particularly in the immediate weeks to months after the event.^1^ Recurrent events are most commonly the result of additional plaque rupture or erosion and are associated with significant morbidity and mortality.^2,3^ Although they may occur at the site of the index MI vessel, they are equally likely to occur at a different site anywhere in the coronary artery tree.^2^ Although a low level of high-density lipoprotein (HDL) cholesterol is a risk factor for major adverse cardiovascular events (MACE end points),^4–12^ it remains unclear whether raising HDL will reduce MACE end points because several therapies that raised HDL cholesterol were not associated with improved clinical outcomes.^13–17^ These studies may have been limited by the failure to enrich for patients with high modifiable risk, off-target toxicity, or failure to raise functional HDL. Cholesterol efflux capacity, an ex vivo measure of HDL function, evaluates the ability of HDL to remove excess cholesterol from atherosclerotic plaque for transport to the liver. It is a correlate of MACE end points that is independent of HDL cholesterol, and it may be more viable to improve clinical outcomes by identifying pharmacotherapies that act rapidly after acute MI to improve cholesterol efflux and thereby reduce plaque burden and stabilize vulnerable plaque, rather than raising HDL alone.^18–20^ It is important to note that the majority of the failed HDL cholesterol–raising trials evaluated long-term pharmacotherapy, and therapy was not initiated in the immediate post-MI period, a time when cholesterol efflux is significantly impaired.^21–23^

CSL112 is a plasma-derived apolipoprotein A-I (apoA-I), the primary functional component of HDL, reconstituted into disk-shaped lipoproteins with phosphatidylcholine and stabilized with sucrose.^24^ Initial studies of CSL112 have demonstrated a significant dose-dependent increase in plasma apoA-I and a dose-dependent increase in total and ATP-binding cassette A1 (ABCA1)–dependent cholesterol efflux capacity.^25–27^ A favorable safety profile has been demonstrated in the clinical program to date, including in patients with stable atherosclerotic disease, although it has not been characterized in patients with acute MI.^27^ A prototype formulation of CSL112 was discontinued from development because of the occurrence of transient elevations of hepatic enzymes presumed to be related to the phosphatidylcholine excipient content.^28,29^ Risk of renal toxicity has been described with high doses of intravenous sucrose. We therefore assessed both hepatic and renal function after infusion of this lower-phosphatidylcholine– and low-sucrose–containing preparation of CSL112 in patients with MI.

The AEGIS-I trial (Apo-I Event Reducing in Ischemic Syndromes I) was a multicenter, randomized, placebo-controlled, dose-ranging phase 2b clinical trial that primarily aimed to assess safety and tolerability and secondary and exploratory objectives including time to first occurrence of MACE end points and the pharmacokinetics and pharmacodynamics of 4 weekly administrations of 2 doses of CSL112 compared with placebo among patients with acute MI and either normal renal function or mild renal impairment (ClinicalTrials.gov; NCT02108262).

## Methods

### Study Oversight

AEGIS-I was a randomized, double-blind, placebo-controlled, dose-ranging phase 2b trial designed in collaboration between the study sponsor (CSL Behring) and members of the executive and steering committee (online-only Data Supplement). Statistical analyses were conducted independently by the PERFUSE Study Group (Perfusion Use in Stroke Evaluation Study) using the Study Data Tabulation Model data sets. The executive committee drafted all versions of the manuscript and agreed to the content of the final version. The sponsor had the opportunity to review and comment on the final draft of the manuscript but had no editorial authority. The study design was in accordance with the 1964 Declaration of Helsinki and its later amendments and was approved by the appropriate national and institutional regulatory agencies and ethics committees. An independent data and safety monitoring board (online-only Data Supplement) monitored the trial and reviewed unblinded data.

### Study Population

Men and women at least 18 years of age with a clinical presentation consistent with a type I (spontaneous) MI within the past 7 days who had either normal renal function or mild renal impairment were enrolled. The criteria for MI were based on the third universal definition of MI.^30^ *Normal renal function* was defined as an estimated glomerular filtration rate ≥90 mL·min^−1^·1.73 m^−2^, and *mild renal impairment* was defined as estimated glomerular filtration rate <90 and ≥60 mL·min^−1^·1.73 m^−2^.

Major exclusion criteria included evidence of current hepatobiliary disease, baseline moderate or severe chronic kidney disease, history of contrast-induced acute kidney injury, or ongoing hemodynamic instability. Among subjects who underwent angiography and were administered a contrast agent, stable renal function at least 12 hours after contrast administration (ie, no increase in serum creatinine ≥0.3 mg/dL from the precontrast value) was required for enrollment. A full list of inclusion and exclusion criteria is provided in the online-only Data Supplement. An institutional review committee approved the study, and all subjects were provided written informed consent before enrollment.

### Study Protocol

The US Food and Drug Administration mandated a review of renal and hepatic safety by the data and safety monitoring board after the first 9 patients were enrolled, and after data and safety monitoring board approval, enrollment in the main study was initiated. Eligible patients were first stratified by renal function (either normal renal function or mild renal impairment) and were then randomly assigned with a 1:1:1 ratio to 1 of 3 treatment groups: low-dose CSL112 (2 g apoA-I per dose), high-dose CSL112 (6 g apoA-I per dose), or placebo. The study drug was administered as a weekly 2-hour intravenous infusion for 4 consecutive weeks (on study days 1, 8, 15, and 22; online-only Data Supplement). The active treatment period was defined as the time from the administration of the first dose of study drug (study day 1) until 1 week after the last infusion (study day 29). All patients were to complete the safety follow-up period on study day 112 (end of study visit).

Patients were routinely evaluated at predetermined intervals from screening until the final follow-up visit. Evaluations included physical examinations, serum creatinine, total bilirubin, alkaline phosphatase, alanine transaminase, aspartate transaminase, blood urea nitrogen, serum creatine, glucose, metabolic, cardiovascular and lipid biomarkers, markers of immunogenicity, and assessments of infusion site, bleeding, and adverse events. The occurrence of MACE end points was also monitored for all subjects for up to 1 year after randomization or until the last randomized subject completed the study day 112 visit.

Plasma concentrations of apoA-I and ex vivo cholesterol efflux were measured at several time points. In addition, a pharmacokinetics/pharmacodynamics substudy was conducted among 63 patients. Subjects included in the substudy were equally stratified by renal function and were randomly assigned with a ratio of 2:3:3 to placebo, low-dose CSL112 (2 g apoA-I per dose), or high-dose CSL112 (6 g apoA-I per dose), respectively. The ability of plasma to mediate cholesterol efflux from cultured J774 cells was measured as previously described.^26^ These assays measure both total cholesterol efflux capacity and the efflux that may be attributed to the ABCA1 transporter. Both efflux measures are presented as percent of cellular cholesterol content. Additional details of the AEGIS-I trial design have previously been published.^31^

### Coprimary Safety End Points

The coprimary safety end points were rates of hepatotoxicity and renal toxicity. Hepatotoxicity was defined as the incidence of either alanine transaminase >3 times the upper limit of normal or total bilirubin >2 times the upper limit of normal that was confirmed on repeat measurement. Renal toxicity was defined as either a serum creatinine ≥1.5 times the baseline value that was confirmed on repeat measurement or a new-onset requirement for renal replacement therapy. Both hepatic and renal safety end points were evaluated from baseline (before the first infusion) through the end of the active treatment period (study day 29). All measures for the coprimary safety end points were based on central laboratory values.

### Secondary and Exploratory End Points

Secondary and exploratory efficacy end points were assessed in the intent-to-treat population (all patients randomized, including those who did not receive study drug) and included the time to first occurrence of a MACE, which was defined as the composite of cardiovascular death, nonfatal MI, ischemic stroke, or hospitalization for unstable angina, from randomization until the last treated subject completed study day 112. An independent clinical events committee that was blinded to treatment assignment adjudicated all MACE end points.

Bleeding was assessed as a secondary safety end point because the majority of subjects were anticipated to be treated with dual antiplatelet therapy after MI. Measured and baseline-corrected plasma apoA-I concentrations, pharmacodynamic characteristics of CSL112, including changes in total and ABCA1-dependent cholesterol efflux measures (ex vivo), and lipid, metabolic, and cardiovascular biomarkers were assessed. Additional prespecified end points have previously been described.^31^

### Statistical Analysis

Statistical analyses were conducted with SAS version 9.4. All safety end points were evaluated in the safety population, which consisted of randomized subjects who received at least 1 partial dose of the study drug. In the safety population, subjects were classified according to the actual treatment they received and their true renal stratum. Efficacy end points were evaluated in the intent-to-treat population, which consisted of all randomized subjects. In the intent-to-treat population, subjects were classified according to the treatment they were randomized to and according to the renal function stratum they were randomized from, regardless of actual treatment or true renal function stratum. Additional populations such as the pharmacokinetics analysis population, pharmacokinetics/pharmacodynamics analysis population, and biomarker analysis population were predefined in the study protocol.

The Newcombe-Wilson score method was used to calculate the 2-sided 95% confidence intervals of the difference in rates (CSL112 minus placebo) for the coprimary safety end points. The upper bound of the 2-sided 95% confidence interval was specified for testing the coprimary end points, comparing with the specified thresholds for hepatic and renal end points for the noninferiority assessment. This gives a 1-sided 2.5% type I error for each of the hepatic and renal end points and was based on an application of the Bonferroni method to control the overall type I error at 5%. Noninferiority criteria were prespecified to be met for the rate difference if the upper bound of the 95% confidence interval was ≤4% in hepatic outcomes and ≤5% in renal outcomes for a pairwise treatment group comparison. Bleeding rates were compared among the 3 groups. Adverse events are presented with the use of descriptive statistics in the online-only Data Supplement.

Although not powered to detect differences in MACE end points, secondary and exploratory MACE outcomes were evaluated by calculating differences in time to first MACE between the treatment groups with a Cox proportional hazards model, with treatment assignment and baseline renal function stratum as covariates. A 2-sided log-rank test *P* value was calculated for each CSL112 dose versus placebo with stratification by renal function. No formal hypothesis testing for MACE end points was intended.

## Results

**Figure 1.**
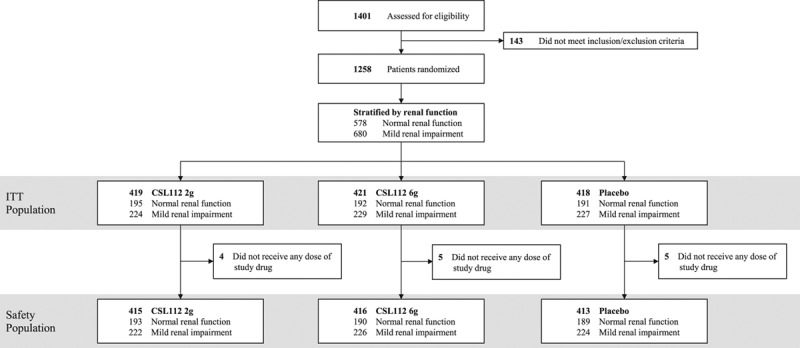
**CONSORT (Consolidated Standards of Reporting Trials) diagram.** ITT indicates intent to treat.

From January 2015 through November 2015, a total of 1258 patients in 16 countries were randomized, of whom 1244 (99.6%) received at least 1 dose of study drug and 1147 (91.2%) received all 4 infusions. A total of 680 patients (54.1%) were stratified to the normal renal function stratum, and 578 (45.9%) were stratified to the mild renal impairment stratum (Figure 1). For the index event, 61.6% of patients experienced ST-segment–elevation MI and 38.4% experienced non–ST-segment–elevation MI. The median duration from the index event to randomization was 4 days, and although 24 to 34 patients per treatment group had 1 year of follow-up, the median duration of follow-up was 7.5 months (interquartile range, 5.8–9.7 months). Baseline characteristics were well balanced among the 3 treatment groups (Table 1).

**Table 1.**
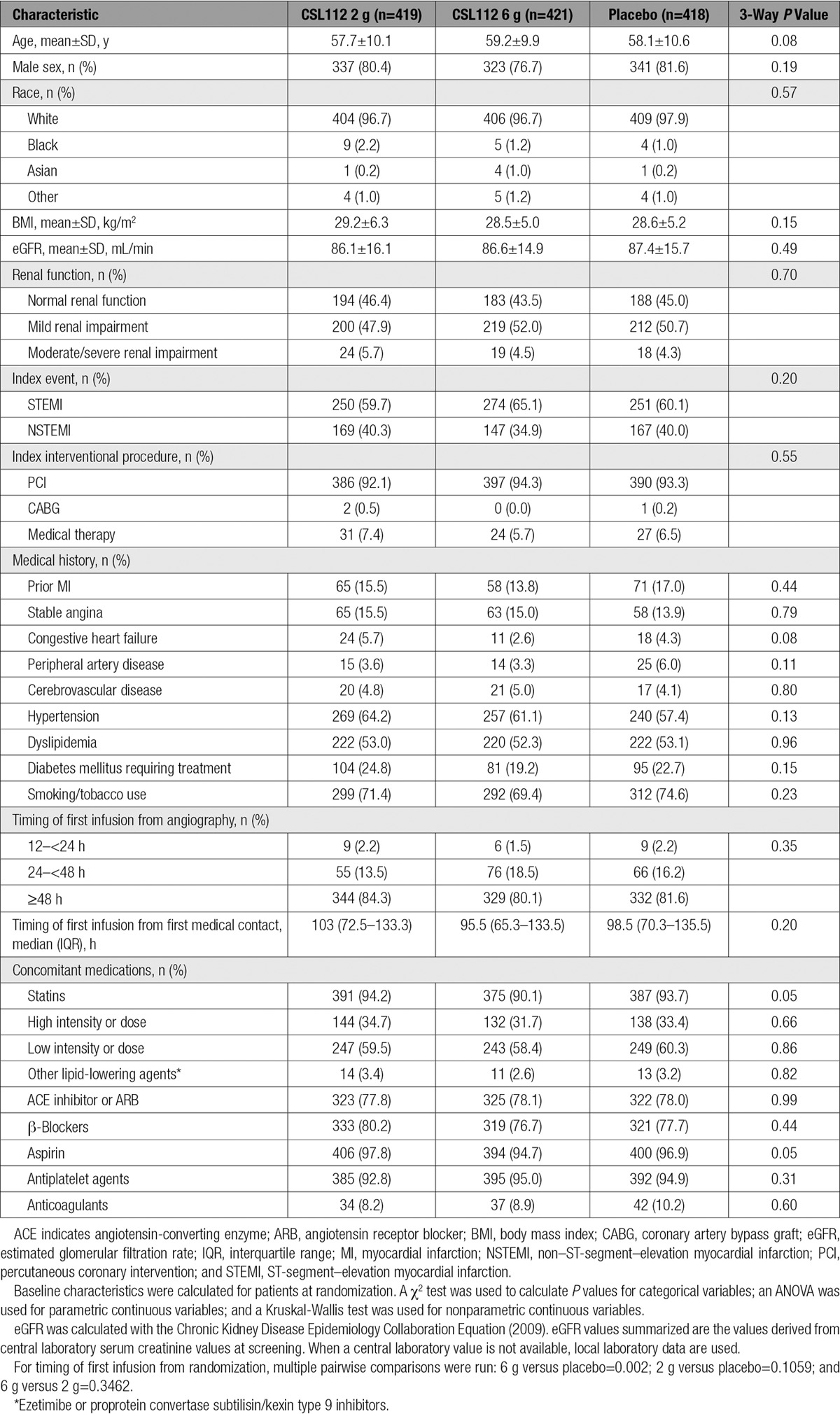
Baseline Characteristics

### Coprimary End Points Results

During the active treatment period, the coprimary safety end point of hepatic impairment occurred in 0 patients (0.0%) in the placebo group, 4 of 415 patients (1.0%) in the 2-g dose group (*P*=0.12 versus placebo), and 2 of 416 patients (0.5%) in the 6-g dose group (*P*=0.50 versus placebo). Both dose comparisons with placebo were not significantly different and were within the prespecified margin of ≤4% (Table 2). There were no Hy law cases (ie, concomitant elevation of alanine transaminase/aspartate transaminase and bilirubin with no other reason to explain the combination) in the trial. Results from 2 prespecified sensitivity analyses, including patients with elevated baseline bilirubin and all elevated values regardless of confirmation values, were consistent with the results of the primary safety analysis (Table I in the online-only Data Supplement).

**Table 2.**
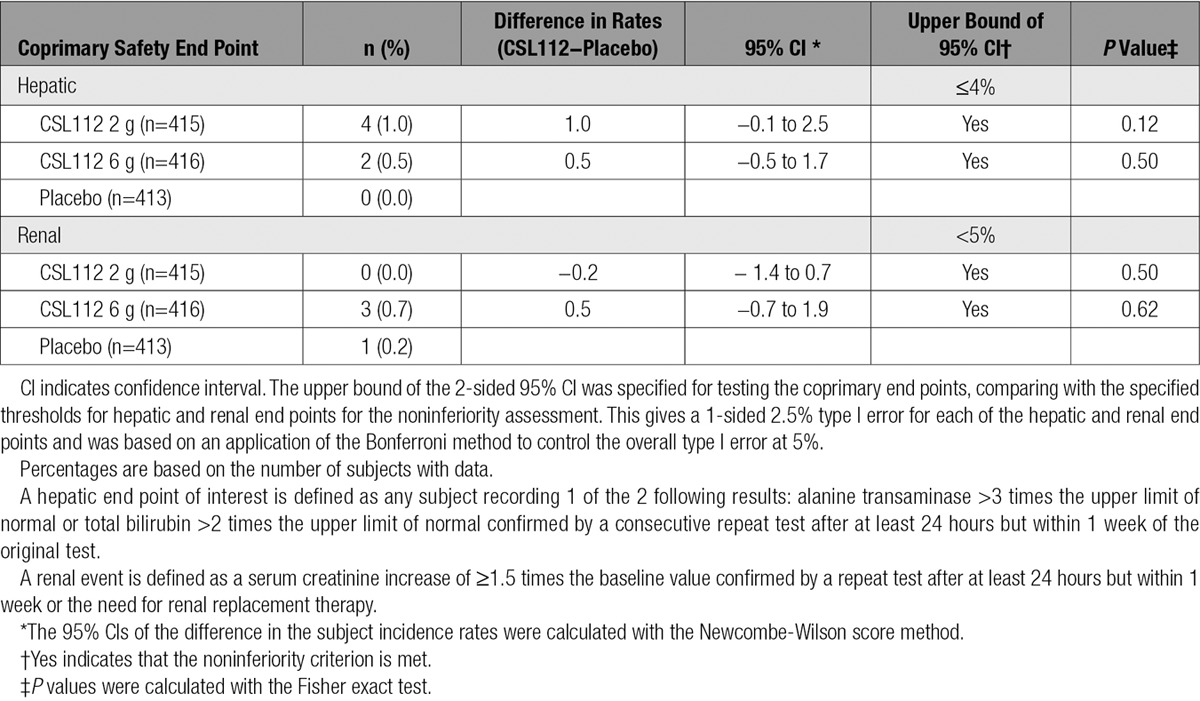
Coprimary Safety End Points

The coprimary safety end point of renal impairment occurred in 1 of 413 patients (0.2%) in the placebo group, 0 of 415 patients (0.0%) in the 2-g dose group (*P*=0.50 versus placebo), and 3 of 416 patients (0.7%) in the 6-g dose group (*P*=0.62 versus placebo). Both dose comparisons with placebo were not significantly different and were within the prespecified margin of ≤5% (Table 2). Additional prespecified exploratory safety analyses and post hoc analyses are shown in Tables II and III in the online-only Data Supplement.

### Secondary and Exploratory End Points Results

**Figure 2.**
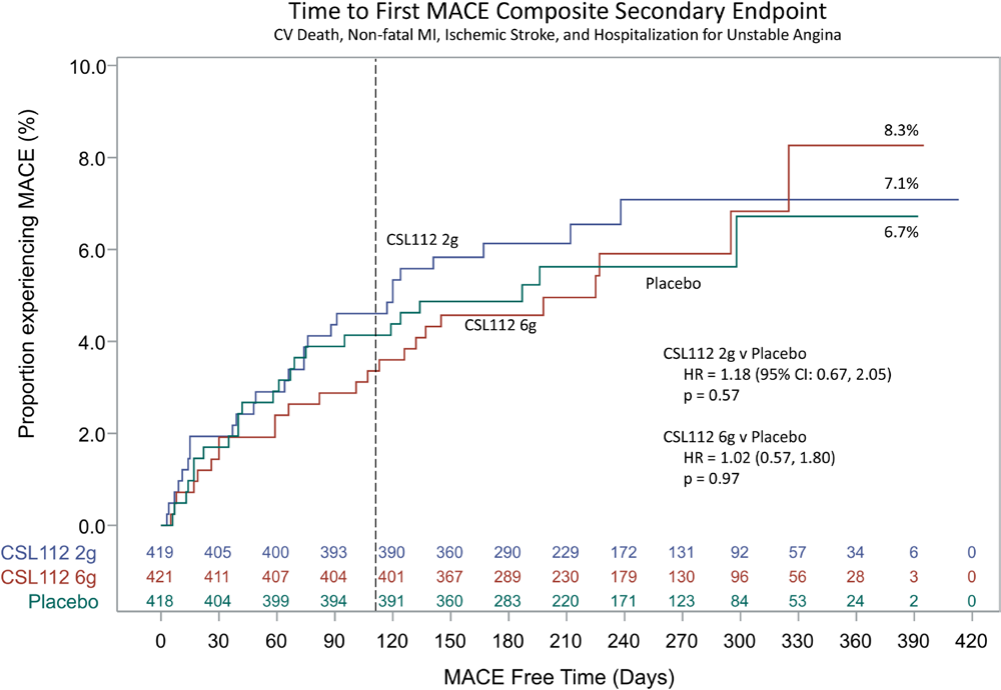
**Time to occurrence of first major adverse cardiovascular event (MACE).** Composite of cardiovascular (CV) death, nonfatal myocardial infarction (MI), ischemic stroke, and hospitalization for unstable angina. HR indicates hazard ratio.

**Figure 3.**
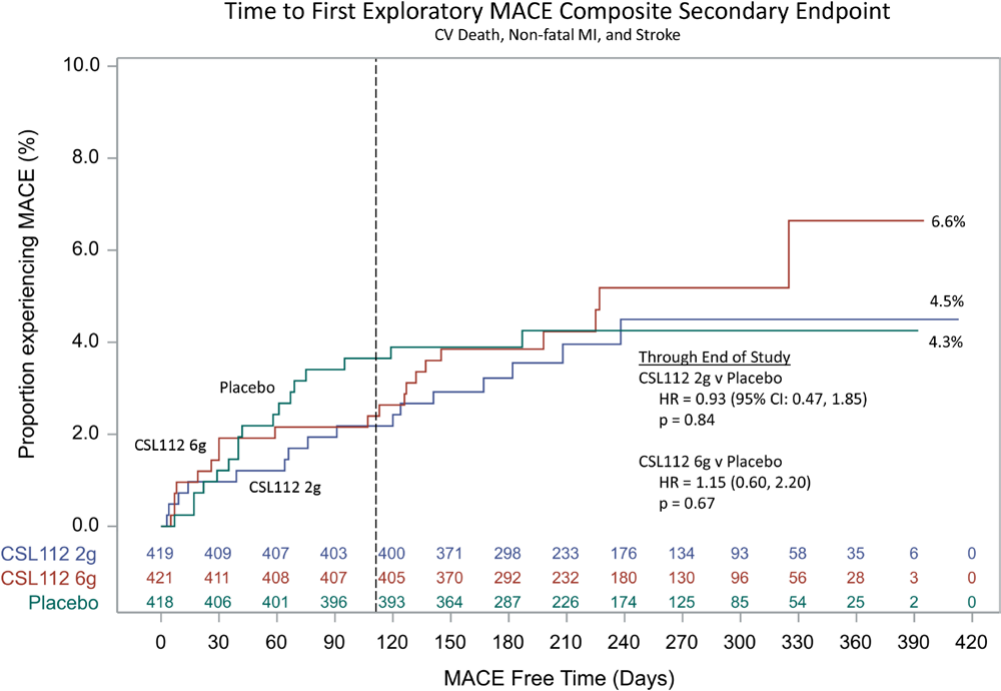
**Time to occurrence of first exploratory major adverse cardiovascular event (MACE).** Composite of cardiovascular (CV) death, nonfatal myocardial infarction (MI), and stroke. HR indicates hazard ratio.

Through 12 months of follow-up, the risk of the MACE composite secondary end point (cardiovascular death, nonfatal MI, ischemic stroke, and hospitalization for unstable angina) with CSL112 therapy compared with placebo was similar (low dose [2 g], 27 of 419 [6.4%] versus placebo, 23 of 418 [5.5%]; hazard ratio, 1.18; 95% confidence interval, 0.67–2.05; *P*=0.72; and high dose [6 g], 24 of 421 [5.7%]; hazard ratio, 1.02; 95% confidence interval, 0.57–1.80; *P*=0.52; Figure 2). Similar risks among treatment groups for the exploratory MACE composite end points were observed, including in the traditional phase 3 end point of cardiovascular death, nonfatal MI, and stroke (Figure 3). As for the secondary MACE composite end point, the majority of additional exploratory MACE end points were similar among treatment groups. There was a difference in the number of cardiovascular-related deaths when CSL112 6-g apoA-I (n=4 [1.0%]; *P*=0.0477) was compared with placebo (n=0 [0.0%]), but this was not seen when CSL112 2g apoA-I (n=2 [0.5%]; *P*=0.32) was compared with placebo. However, the number of patients experiencing cardiovascular-related deaths was low (Table 3). Similarly, a difference in the number of heart failure events was observed when CSL112 6-g apoA-I (n=4, 1.0%; *P*=0.2525) was compared with placebo (n=1, 0.2%) and CSL112 2g apoA-I (n=5, 1.2%; *P*=0.1205) was compared with placebo. The number of patients experiencing heart failure was low (Table 3).

**Table 3.**
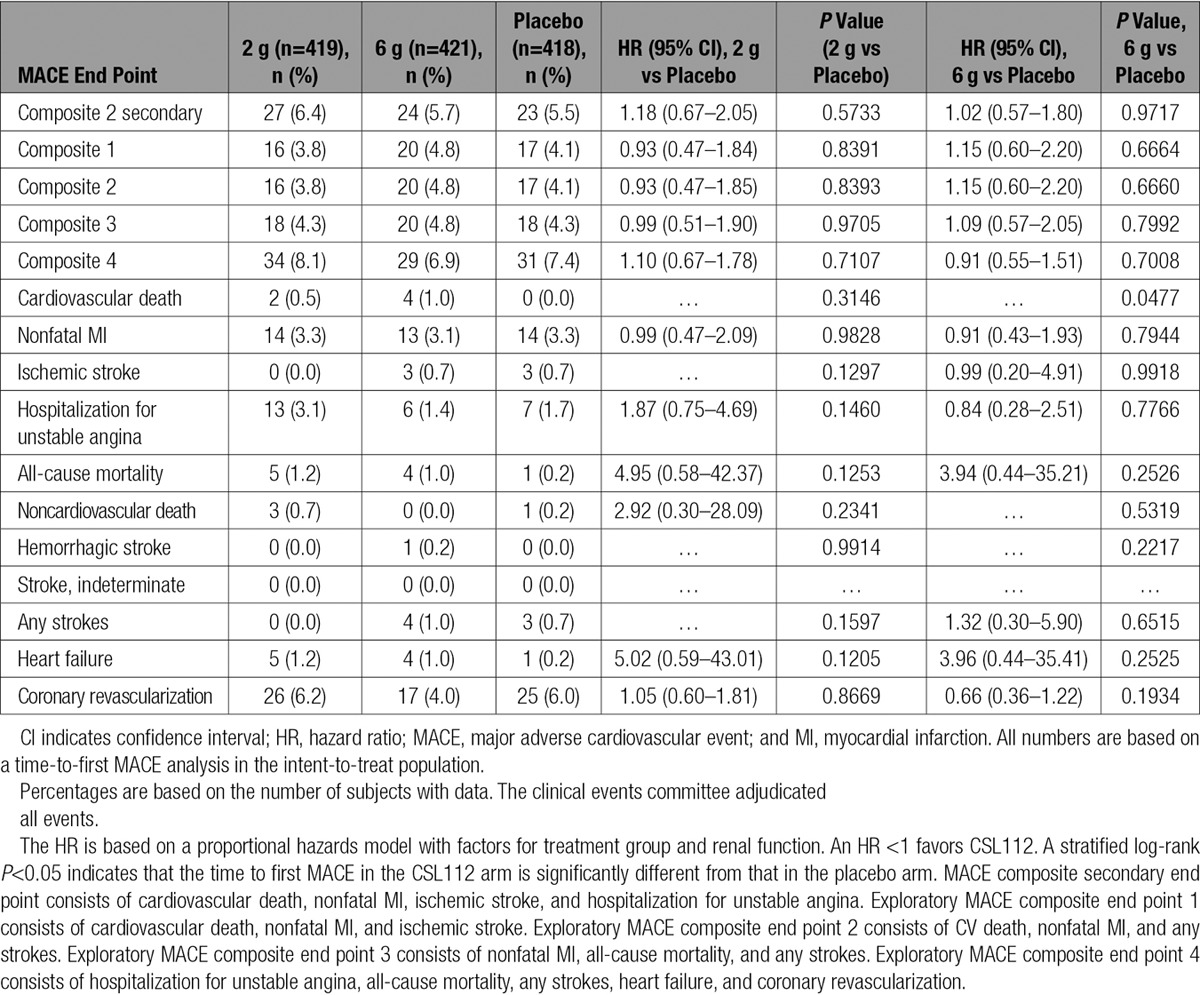
MACE End Points in the Intent-to-Treat Population

The rates of all grades of Bleeding Academic Research Consortium bleeding were low and comparable among the 3 arms (Table 4). Drug hypersensitivity reactions and infusion site reactions were well balanced across groups. Overall, the rates of serious and life-threatening adverse events and serious adverse events leading to drug discontinuation were relatively low and comparable across all groups (Tables IV and V in the online-only Data Supplement).

**Table 4.**
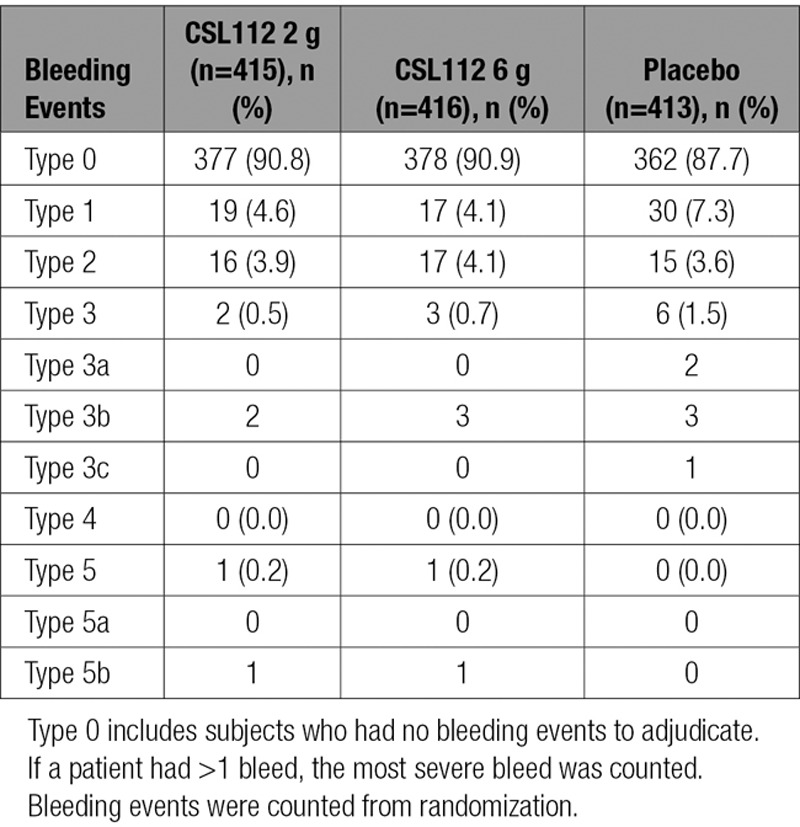
Bleeding Academic Research Consortium Evaluation Grades for Worst Bleeding Events: Safety Population

Baseline plasma concentrations of apoA-I, cholesterol efflux capacity, and lipid and cardiovascular biomarkers were similar among the 3 treatment groups (Table 5). Infusion of CSL112 caused a dose-dependent elevation of both apoA-I and cholesterol efflux capacity (Table 6). The 2-g dose elevated apoA-I 1.29-fold and total cholesterol efflux capacity 1.87-fold, whereas the 6-g dose elevated apoA-I 2.06-fold and total cholesterol efflux capacity 2.45-fold. Consistent with prior findings, the elevation of ABCA1-dependent cholesterol efflux capacity (3.67-fold for the 2-g dose, 4.30-fold for the 6-g dose) was substantially greater than the elevation of either apoA-I or total cholesterol efflux capacity, suggesting that CSL112 may increase not only the amount of circulating apoA-I but also the activity for ABCA1-dependent efflux on a per–apoA-I basis.^26^ We assessed this “specific activity” of the circulating apoA-I pool for ABCA1-dependent cholesterol efflux capacity by calculating the ABCA1-dependent cholesterol efflux capacity/apoA-I ratio at the end of the infusion. Infusion of CSL112 caused a 2.51-fold increased ratio for the 2-g dose group (0.05) and a 1.78-fold increased ratio for the 6-g dose group (0.035) compared with the placebo group (0.02).^26^ The elevation in ABCA1-dependent efflux capacity was greater than the elevation of apoA-I. Although this ratio is not a validated measure, it could be speculated that the infusion elevates not just the quantity but also the functionality of the apoA-I pool. Indeed, the ABCA1-dependent cholesterol efflux capacity/apoA-I ratios were elevated with both doses of CSL112 compared with placebo (Table III in the online-only Data Supplement)

**Table 5.**
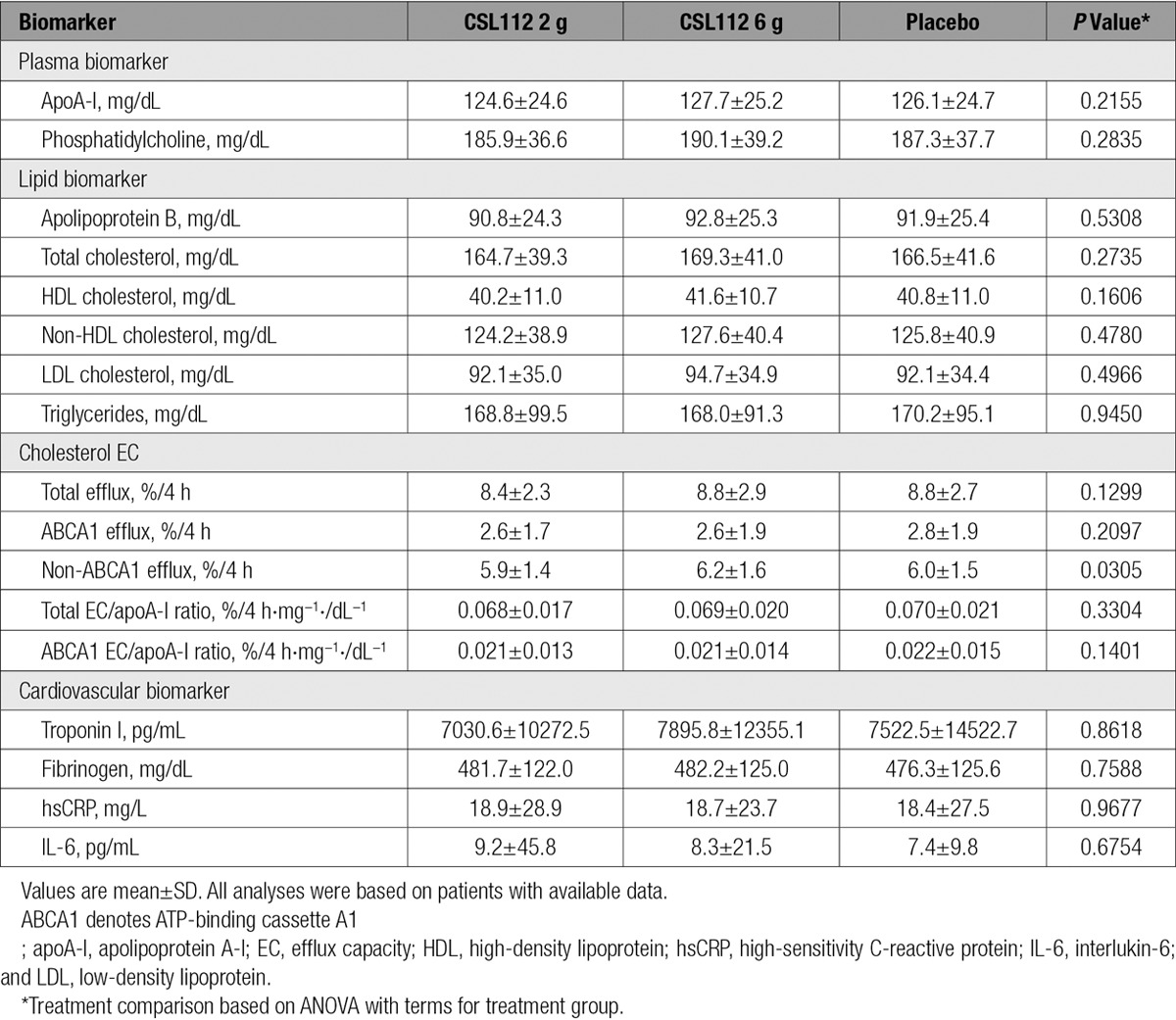
Baseline Lipid and Cardiovascular Biomarkers

**Table 6.**
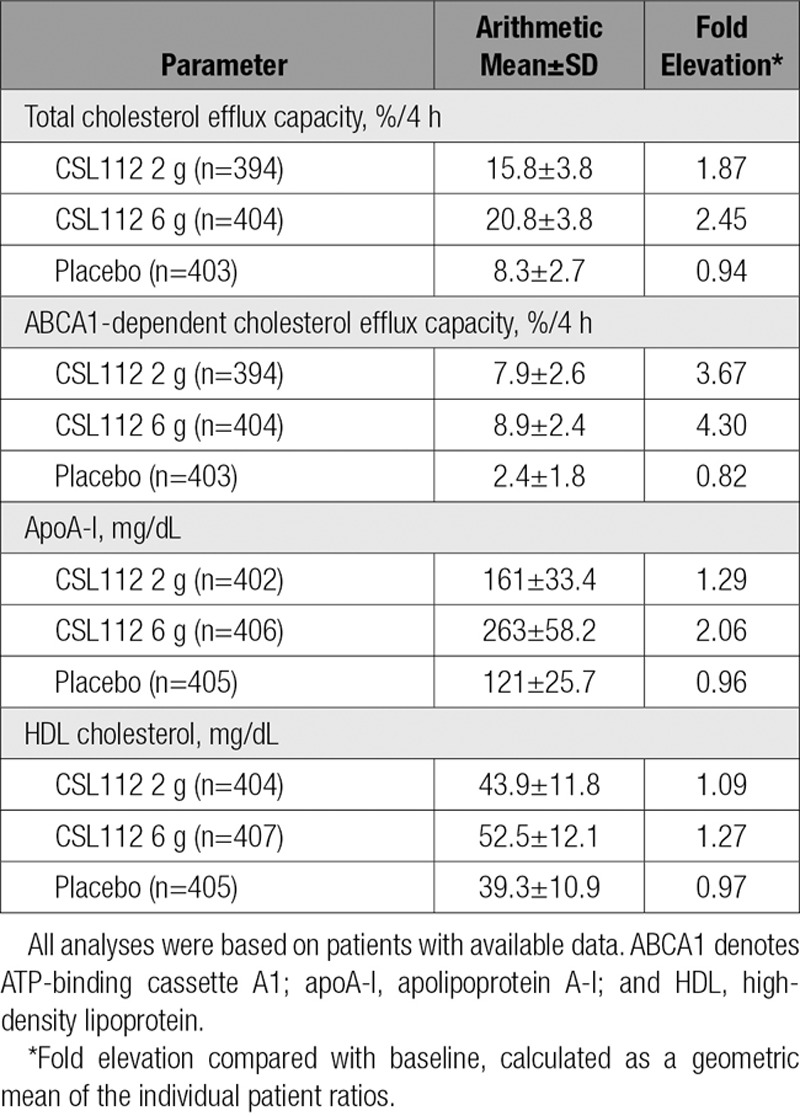
Cholesterol Efflux, HDL Cholesterol, and ApoA-I Values Immediately After Infusion of CSL112

## Discussion

Infusions of CSL112, a reconstituted plasma-derived apoA-I, at both low (2 g) and high (6 g) doses administered as 4 weekly infusions beginning within 7 days of acute MI were not associated with alterations in either liver or kidney function. This was the first study in which CSL112 was administered to patients with acute MI and the first study in which it was added to acute MI standard of care. Establishing safety and feasibility in the acute MI setting was important before the initiation of a large-scale phase 3 outcomes trial. The results from AEGIS-I suggest that the current formulation of CSL112 compared with the prototype formulation did not demonstrate a hepatic safety concern. Furthermore, infusion of CSL112 shortly after a contrast load among patients with MI was not associated with renal toxicity, demonstrating the feasibility of administering CSL112 to patients with MI with normal renal function or mild renal impairment shortly after angiography. A study in patients with MI with moderate renal impairment is ongoing.

The number of MACE end points overall was low (n=74), as was the number of subjects with complete follow-up through 1 year (89 of 1258). The statistical power to assess the secondary MACE end point was very low, ≈8.4% (Table VII in the online-only Data Supplement). MACE rates were generally comparable between groups, although cardiovascular mortality was higher in the 6-g group compared with the placebo group (4 versus 0 deaths; *P*=0.0477). The calculated *P* value was not adjusted for the multiplicity of 32 efficacy comparisons. There was no clustering of death in proximity to the CSL112 infusion (Table VI and Figure I in the online-only Data Supplement). It should be noted that indeterminant causes of death were included as cardiovascular death. The isolated difference in mortality was inconsistent with the overall similarity in MACE rates.

Compared with placebo, CSL112 was also associated with an improvement in measures of cholesterol efflux capacity. It has been postulated that improvements in HDL function, rather than HDL concentration, may be more important for the stabilization of atherosclerotic plaque lesions and the reduction of cardiovascular events. In the Dallas Heart Study, high cholesterol efflux capacity, a marker of effective reverse cholesterol transport, was associated with a 67% lower risk of MACE end points compared with low cholesterol efflux capacity,^18^ an association that was independent of HDL concentrations. To date, although HDL-raising therapies have indeed increased HDL concentrations, they have had a modest or no effect on cholesterol efflux, a finding that may explain at least in part why HDL-raising therapies have failed to reduce MACE outcomes in the past.^32–37^ In contrast, cholesterol efflux capacity was markedly elevated immediately after CSL112 infusion. In particular, ABCA1-dependent efflux, a pathway especially relevant to cholesterol-laden cells in plaque, was elevated >3-fold after infusion of CSL112. It is noteworthy that the elevation in the ABCA1-dependent efflux capacity was greater than the elevation of apoA-I, thus suggesting that infusion elevates not just the quantity but also the functionality of the apoA-I pool. Indeed, the ABCA1-dependent cholesterol efflux capacity/apoA-I ratios were elevated with both doses of CSL112 compared with placebo (Table 6). Prior mechanistic studies^38^ have shown comparable functional changes and have determined that CSL112 elevates ABCA1-dependent efflux by remodeling endogenous HDL to form smaller, more functional HDL species with a high ability to interact with ABCA1.

The elevation of cholesterol efflux caused by CSL112 has been shown to be transient and recedes to baseline with clearance of the apoA-I.^26^ It is not known how a transient enhancement of cholesterol efflux capacity immediately after acute MI will affect clinical outcomes compared with the sustained or long-term measures of cholesterol efflux assessed in the Dallas Heart Study.^18^ Although MACE end points were not reduced in AEGIS-I, this phase 2b study was designed as a safety trial and was not sufficiently powered to assess efficacy (Table VII in the online-only Data Supplement). Consistent with other phase 2 safety studies, MACE end points were explored in AEGIS-I to assess the timing and frequency of events and to identify subgroups of patients at higher risk of events so that an adequately powered phase 3 study could be planned to definitively assess the efficacy. Although these analyses are exploratory, they were prespecified so as to focus the analyses for phase 3 planning.

### Limitations

The coprimary safety end points were less frequent than anticipated for the noninferiority analysis, but the very low frequency of these events suggests that there is not a clinically relevant hepatic or renal safety signal. Although several lipid and lipoprotein analyses were performed, lipoprotein(a) and apolipoprotein E were not assessed after infusion.

This phase 2 safety study was underpowered to assess efficacy and was not designed to test for efficacy. For the secondary MACE end point, the power was 8.4% to detect a clinically relevant 15% risk reduction assuming a placebo event rate of 5.5% (Table VII in the online-only Data Supplement). The statistical power of other end points can be found in the online-only Data Supplement. As were many phase 2 studies, this trial was undertaken primarily to assess safety, tolerability, pharmacokinetics, and pharmacodynamics.

### Conclusions

Four weekly infusions of CSL112, a reconstituted plasma-derived apoA-I, at both low (2 g) and high (6 g) doses beginning within 7 days of acute MI and in proximity to contrast media administration were feasible, were not associated with alterations in either liver or kidney function or other significant safety concern, and were associated with immediate enhancements in cholesterol efflux capacity. Further assessment of the clinical efficacy of CSL112 for the reduction of early recurrent cardiovascular events after acute MI is warranted in an adequately powered, multicenter, randomized phase 3 trial.

## Sources of Funding

This study was funded by the sponsor, CSL Behring LLC.

## Diclosures

All authors have received research grant support from CSL Behring. Drs D’Andrea and Deckelbaum are employees of the sponsor of the trial, CSL Behring. Dr Gibson and spouse, Dr Alexander, and Dr Tricoci received consulting monies from CSL Behring. Dr Steg has research grants from Merck, Sanofi, and Servier and speaking or consulting fees from Amarin, Amgen, AstraZeneca, Bayer, Boehringer-Ingelheim, Bristol-Myers-Squibb, CSL Behring, Daiichi-Sankyo, GlaxoSmithKline, Janssen, Lilly, Merck Novartis, Pfizer, Regeneron, Sanofi, Servier, and The Medicines Company. Dr Harrington has research grants and contracts from the National Heart, Lung, and Blood Institute, *Patient-Centered Outcomes Research Institute*, Duke, Harvard, Astra, CSL, GlaxoSmithKline, Janssen, Merck, Novartis, Portola, Sanofi-Aventis, and The Medicines Company; has received consulting and advisory fees from Adverse Events, Amgen, Element Science, Gilead, Merck, MyoKardia, The Medicines Company, Vida Health, and WebMD; and is on the board of directors for the American Heart Association, SHC, Scanadu (mobile health), and SignalPath (software).

## Supplementary Material

## Clinical Perspective

### What Is New?

- Among patients with acute myocardial infarction, 4 weekly infusions of a reconstituted infusible human apolipoprotein A-I, CSL112, are associated with a dose-dependent elevation of circulating apolipoprotein A-I and cholesterol efflux capacity without adverse hepatic or renal outcomes.

### What Are the Clinical Implications?

- Further studies are warranted to assess the clinical benefit of CSL112 in reducing cardiovascular events after acute myocardial infarction.
