# Supplementary material for: Safety and Tolerability of CSL112, a Reconstituted, Infusible, Plasma-Derived Apolipoprotein A-I, After Acute Myocardial Infarction: The AEGIS-I Trial (ApoA-I Event Reducing in Ischemic Syndromes I)
Source: Circulation. 2016 Dec 12;134(24):1918–30. doi: 10.1161/CIRCULATIONAHA.116.025687 (PMC5147036; doi:10.1161/CIRCULATIONAHA.116.025687)
Supplement: Supplementary file 1 [file cir-134-1918-s001.pdf]

SUPPLEMENTARY MATERIAL

SUPPLEMENTARY APPENDIX

This appendix has been provided by the authors to give readers additional information about their work.
Supplement to: [Name of manuscript]
(PDF updated [Date])

Table of Contents

Table S1 Sensitivity Analyses of Co-Primary Safety Endpoints..4
Table S2 Post-hoc Sensitivity Analysis of Co-Primary Endpoints with Bonferroni Adjustment for Multiple Treatment Comparisons ……………………………………………………….………5
Table S3 Cholesterol Efflux and apoA-I ratios immediately after infusion of CSL112……………6
Table S4 Treatment Emergent Adverse Events, Frequency of Events: Safety Population………..7
Table S5 Treatment Emergent Adverse Events, Percentage of Patients: Safety Population……...8
Table S6 Summary of Fatal Outcomes by Study Period………………………………………..9
Table S7 Sample size and power calculation of MACE endpoints required to detect a ≥ 15% risk reduction at a two-sided significance level of 0.05………………………………………..……….10
Figure S1 Days from Randomization until Death……………………………………………………11
Appendix A – Committees, Leadership and Other Collaborations…………………………………..12
AEGIS-I Executive Committee... 11
AEGIS-I Steering Committee 11
PERFUSE Study Group.. 11
CSL Behring, King of Prussia, Pennsylvania (Sponsor) 11
Independent Statistical Groups 11
Data and Safety Monitoring Board... 12
Clinical Events Committee.. 123
Core Laboratories... 123
North America Site Management and Monitoring... 123
Contract Research Organization.. 123
Appendix B - Enrolling Sites.. 134

Table S1
Sensitivity Analyses of Co-Primary Safety Endpoints

Co-Primary Safety Endpoint	n (%)	Difference in rates (CSL11 – placebo)	95% CI [a]	Upper Bound of 95% CI [b]	p-value [c]
Hepatic – No Confirmatory Result				≤ 4%	
CSL112 2g (N=415)	9 (2.2%)	-0.5	(-2.8, 1.7)	Yes	0.64
CSL112 6g (N=416)	5 (1.2%)	-1.5	(-3.6, 0.5)	Yes	0.13
Placebo (N=413)	11 (2.7%)				
Renal – No Confirmatory Result				< 5%	
CSL112 2g (N=415)	4 (1.0%)	-0.5	(-2.3, 1.2)	Yes	0.55
CSL112 6g (N=416)	7 (1.7%)	0.2	(-1.7, 2.1)	Yes	0.79
Placebo (N=413)	6 (1.5%)				

CI=Confidence Interval.

[a] 95% confidence intervals of the difference in the subject incidence rates are calculated using the Newcombe-Wilson score method.

[b] Yes indicates non-inferiority criterion is met.

[c] *P values were calculated using Chi-Square test or Fisher's exact test when expected cell counts were < 5.

* Percentages are based on the number of subjects with data.

* For this sensitivity analysis, a hepatic endpoint of interest is defined as any subject recording one of the two following results: ALT > 3x ULN, Total bilirubin > 2x ULN, without confirmation using a consecutive repeat test after at least 24 hours but within 1 week of original test.

* For this sensitivity analysis, a renal event is defined as a serum creatinine increase of ≥ 1.5X the baseline value or the need for renal replacement therapy, without confirmation using a consecutive repeat test after at least 24 hours but within 1 week of original test.

Table S2
Post-hoc Sensitivity Analysis of Co-Primary Endpoints with Bonferonni Adjustment for Multiple Treatment Comparisons

Co-Primary Safety Endpoint	n (%)	Difference in rates (CSL11 – placebo)	97.5% CI [a]	Upper Bound of 97.5% CI [b]	p-value [c]
Hepatic				≤ 4%	
CSL112 2g (N=415)	4 (1.0%)	1.0	(-0.4, 2.8)	Yes	0.12
CSL112 6g (N=416)	2 (0.5%)	0.5	(-0.8, 2.0)	Yes	0.50
Placebo (N=413)	0 (0.0%)				
Renal				< 5%	
CSL112 2g (N=415)	0 (0.0%)	-0.2	(-1.7, 1.0)	Yes	0.50
CSL112 6g (N=416)	3 (0.7%)	0.5	(-1.0, 2.2)	Yes	0.62
Placebo (N=413)	1 (0.2%)				

CI=Confidence Interval.

[a] The upper bound of the two-sided 95% confidence interval was specified for testing the co-primary endpoints, comparing with the specified thresholds for hepatic and renal endpoints for the non-inferiority assessment. This gives a one-sided 2.5% Type I error for each of the hepatic and renal endpoints and was based on an application of the Bonferroni method to control the overall Type I error at 5%. Multiplicity adjustment was not applied to the two pairwise treatment group comparisons within each co-primary endpoint. This table displays a more conservative assessment using a two-sided 97.5% confidence interval, which further applies a post-hoc Bonferroni adjustment to the treatment group comparisons to achieve an individual one-sided 1.25% Type I error for each of the treatment group comparisons.

[b] Yes indicates non-inferiority criterion is met.

[c] P values were calculated using Fisher's exact test.

Percentages are based on the number of subjects with data.

A hepatic endpoint of interest is defined as any subject recording one of the two following results: ALT > 3x ULN, Total bilirubin > 2x ULN, confirmed by a consecutive repeat test after at least 24 hours but within 1 week of the original test.

A renal event is defined as a serum creatinine increase of ≥ 1.5X the baseline value, confirmed by a repeat test after at least 24 hours but within 1 week, or the need for renal replacement therapy.

Table S3

Cholesterol Efflux and apoA-I ratios immediately after infusion of CSL112

Parameter	Arithmetic Mean ± SD	Fold Elevation
**Total Cholesterol Efflux Capacity/ApoA-I Ratio (%/4 hr/mg/dL)**		
CSL112 2g (N = 394)	0.099 ± 0.023	1.44[@]
CSL112 6g (N = 404)	0.082 ± 0.019	1.18[@]
Placebo (N = 403)	0.069 ± 0.019	-
**ABCA1 Cholesterol Efflux Capacity/ApoA-I Ratio (%/4 hr/mg/dL)**		
CSL112 2g (N = 394)	0.050 ± 0.017	2.51[@]
CSL112 6g (N = 404)	0.035 ± 0.013	1.78[@]
Placebo (N = 403)	0.020 ± 0.014	-

All analyses were based on patients with available data.
@ Fold elevation compared with placebo, calculated as a ratio of the treatment arithmetic means

Table S4
Treatment Emergent Adverse Events, Frequency of Events
Safety Population

Adverse Event (System Organ Class)	CSL112 2g (696)	CSL112 6g (620)	Placebo (639)
Blood & Lymphatic	11 (1.6%)	2 (0.3%)	5 (0.8%)
Cardiac	74 (10.6%)	61 (9.8%)	61 (9.6%)
Congenital, Familial & Genetic	0 (0.0%)	1 (0.2%)	1 (0.2%)
Ear & Labyrinth	2 (0.3%)	7 (1.1%)	6 (0.9%)
Endocrine	1 (0.1%)	1 (0.2%)	5 (0.8%)
Eye	3 (0.4%)	10 (1.6%)	6 (0.9%)
Gastrointestinal	61 (8.8%)	67 (10.8%)	68 (10.6%)
General Disorders & Administration Site Conditions	122 (17.5%)	92 (14.8%)	92 (14.4%)
Hepatobiliary	7 (1.0%)	0 (0.0%)	6 (0.9%)
Immune System	2 (0.3%)	2 (0.3%)	0 (0.0%)
Infections & Infestations	61 (8.8%)	49 (7.9%)	42 (6.6%)
Injury, Poisoning & Procedural Complication	25 (3.6%)	30 (4.8%)	27 (4.2%)
Investigations	39 (5.6%)	54 (8.7%)	57 (8.9%)
Metabolism & Nutrition	24 (3.5%)	10 (1.6%)	21 (3.3%)
Musculoskeletal & Connective Tissue	46 (6.6%)	42 (6.8%)	33 (5.2%)
Neoplasms Benign, Malignant & Unspecified (Incl. Cysts & Polyps)	0 (0.0%)	6 (1.0%)	7 (1.1%)
Nervous System	66 (9.5%)	52 (8.4%)	53 (8.3%)
Product Issues	2 (0.3%)	0 (0.0%)	0 (0.0%)
Psychiatric	14 (2.0%)	9 (1.5%)	10 (1.6%)
Renal & Urinary	16 (2.3%)	6 (1.0%)	15 (2.4%)
Reproductive System & Breast	5 (0.7%)	6 (1.0%)	4 (0.6%)
Respiratory, Thoracic & Mediastinal	63 (9.1%)	52 (8.4%)	58 (9.1%)
Skin & Subcutaneous Tissue	17 (2.4)	24 (3.9%)	17 (2.7%)
Vascular	35 (5.0%)	37 (6.0%)	45 (7.0%)
**Other**			
Adverse event related to study drug?	44 (6.3%)	50 (8.1%)	34 (5.3%)
Adverse event related to study procedure	27 (3.9%)	43 (6.9%)	15 (2.4%)
Adverse events leading to death	3 (0.4%)	2 (0.3%)	1 (0.2%)
Adverse events leading to permanent discontinuation of study drug	11 (1.6%)	8 (1.3%)	9 (1.4%)
Severity of adverse events*			
Grade 1	409 (58.8%)	374 (60.3%)	388 (60.7%)
Grade 2	151 (21.7%)	165 (26.6%)	147 (23.0%)
Grade 3	114 (16.4%)	76 (12.3%)	92 (14.4%)
Grade 4	18 (2.6%)	3 (0.5%)	11 (1.7%)
Grade 5	4 (0.6%)	2 (0.3%)	1 (0.2%)
Serious adverse events	109 (15.7%)	77 (12.4%)	78 (12.2%)
Serious related adverse events	1 (0.8%)	0 (0.0%)	2 (5.9%)

The N's represent the total number of adverse events in each treatment group.

.

Table S5
Treatment Emergent Adverse Events, Percentage of Patients
Safety Population

Adverse Event (System Organ Class)	CSL112 2g (N=415)	CSL112 6g (N=416)	Placebo (N=413)
Blood & Lymphatic	10 (2.4%)	2 (0.5%)	4 (1.0%)
Cardiac	51 (12.3%)	48 (11.5%)	40 (9.7%)
Congenital, Familial & Genetic	0 (0.0%)	1 (0.2%)	1 (0.2%)
Ear & Labyrinth	2 (0.5%)	7 (1.7%)	5 (1.2%)
Endocrine	1 (0.2%)	1 (0.2%)	4 (1.0%)
Eye	3 (0.7%)	6 (1.4%)	4 (1.0%)
Gastrointestinal	42 (10.1%)	42 (10.1%)	46 (11.1%)
General Disorders & Administration Site Conditions	84 (20.2%)	62 (14.9%)	62 (15.0%)
Hepatobiliary	6 (1.5%)	0 (0.0%)	5 (1.2%)
Immune System	2 (0.5%)	2 (0.5%)	0 (0.0%)
Infections & Infestations	47 (11.3%)	39 (9.4%)	38 (9.2%)
Injury, Poisoning & Procedural Complication	18 (4.3%)	17 (4.1%)	24 (5.8%)
Investigations	31 (7.5%)	37 (8.9%)	41 (9.9%)
Metabolism & Nutrition	19 (4.6%)	10 (2.4%)	18 (4.4%)
Musculoskeletal & Connective Tissue	35 (8.4%)	35 (8.4%)	24 (5.8%)
Neoplasms Benign, Malignant & Unspecified (Incl. Cysts & Polyps)	0 (0.0%)	6 (1.4%)	7 (1.7%)
Nervous System	47 (11.3%)	45 (10.8%)	30 (7.3%)
Product Issues	2 (0.5%)	0 (0.0%)	0 (0.0%)
Psychiatric	13 (3.1%)	8 (1.9%)	9 (2.2%)
Renal & Urinary	16 (3.9%)	6 (1.4%)	14 (3.4%)
Reproductive System & Breast	4 (1.0%)	6 (1.4%)	4 (1.0%)
Respiratory, Thoracic & Mediastinal	43 (10.4%)	42 (10.1%)	42 (10.2%)
Skin & Subcutaneous Tissue	14 (3.4%)	22 (5.3%)	17 (4.1%)
Vascular	30 (7.2%)	32 (7.7%)	38 (9.2%)
**Other**			
Study-drug Related adverse events	33 (8.0%)	33 (7.9%)	26 (6.3%)
Adverse events leading to death	3 (0.7%)	2 (0.5%)	1 (0.2%)
Adverse events leading to permanent discontinuation of study drug	11 (2.7%)	8 (1.9%)	9 (2.2%)
Severity of adverse events*			
Grade 1	73 (17.6%)	91 (21.9%)	88 (21.3%)
Grade 2	60 (14.5%)	69 (16.6%)	52 (12.6%)
Grade 3	69 (16.6%)	50 (12.0%)	56 (13.6%)
Grade 4	5 (1.2%)	2 (0.5%)	8 (1.9%)
Grade 5	3 (0.7%)	2 (0.5%)	1 (0.2%)
Serious adverse events	66 (15.9%)	53 (12.7%)	54 (13.1%)
Serious related adverse events	1 (0.2%)	0 (0.0%)	2 (0.5%)

The N’s represent the percentage of patients that experienced an adverse event by treatment group.

*If a patient experienced greater than one adverse event, the most severe was presented for severity of adverse event.

Table S6
Summary of Fatal Outcomes by Study Period

Treatment Period	2g	6g	Placebo
Main Study (N=10)	5	4	1
Active Treatment Period (SD 1-29)	1	2	0
Safety Follow-Up Period (SD 30-112)	2	0	1
MACE Follow-Up Period (SD 113-387)	2	2	0
Safety Lead In (N=1)	1	0	0
Active Treatment Period (SD 1-29)	0	0	0
Safety Follow-Up Period (SD 30-90)	1	0	0

Table S7

Sample size and power calculation of MACE endpoints required to detect a ≥ 15% risk reduction at a two-sided significance level of 0.05

MACE Endpoint	Placebo Event Rate	No. per group required for 90% power	Power with 420 subjects per group
Composite 2° Endpoint	5.5%	14,907	8.4%
Composite 1	4.1%	20,271	7.5%
Composite 2	4.1%	20,271	7.5%
Composite 3	4.3%	19,291	7.7%
Composite 4	7.4%	10,874	9.8%
CV death	0.0%	–	–
Non-fatal MI	3.3%	25,379	7.0%
Ischemic stroke	0.7%	122,620	5.4%
Hosp. for unstable angina	1.7%	50,019	6.0%
All-cause mortality	0.2%	431,171	5.1%
Non-CV death	0.2%	431,171	5.1%
Hemorrhagic stroke	0.0%	–	–
Stroke – indeterminate	0.0%	–	–
Any stroke	0.7%	122,620	5.4%
Heart failure	0.2%	431,171	5.1%
Coronary revascularization	6.0%	13,598	8.8%

Sample size and power were calculated based on the observed event rate in the placebo arm using the Pearson's chi-square test.
For this power calculation both the treatment and placebo arms were standardized to 420 patients.

Figure S1
Days from Randomization until Death

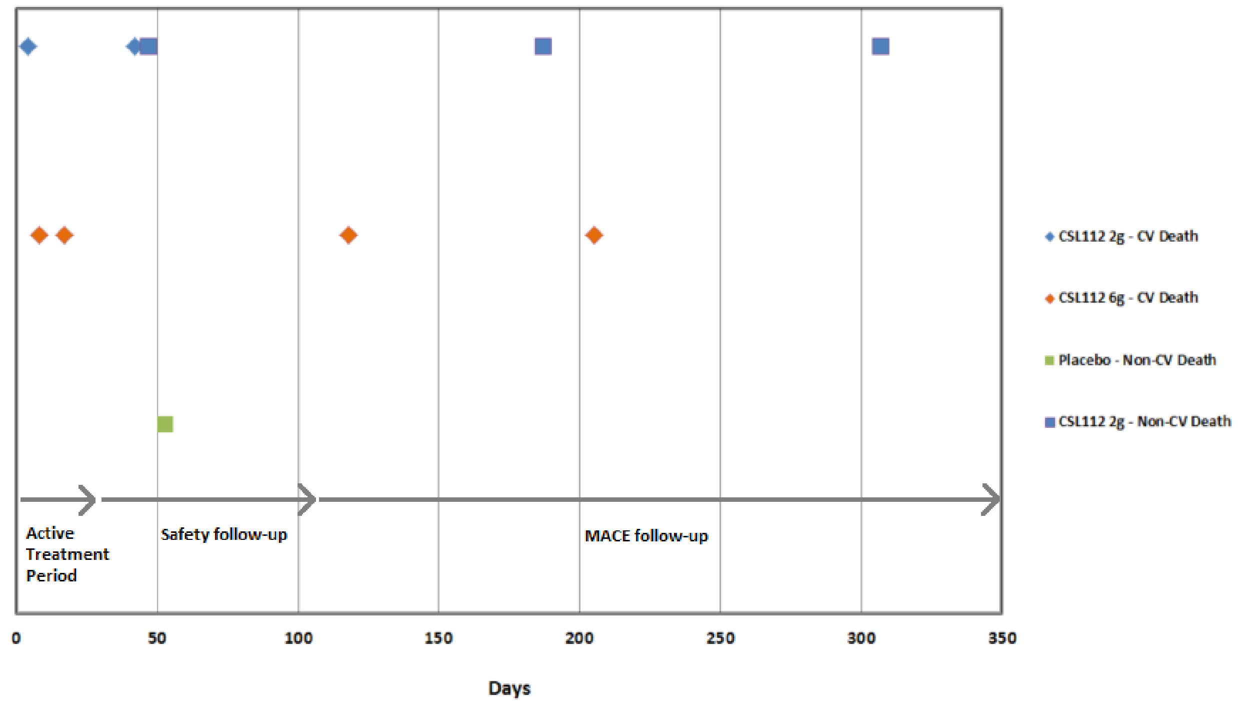

Appendix A – Committees, Leadership and Other Collaborations

AEGIS-I Executive Committee

C. Michael Gibson (Co-Chair), Robert Harrington (Co-Chair), John Alexander, John J. Kastelein, Michael Lincoff, Roxana Mehran, P. Gabriel Steg

AEGIS-I Steering Committee

C. Michael Gibson (Chair), Guiseppe Ambrosio (Italy), Paul Armstrong (Canada), Phil Aylward (Australia), Amadeo Betriu (Spain), Christoph Bode (Germany), Jan Cornel (Netherlands), Anthony Gershlick (United Kingdom), Nina Gotcheva (Bulgaria), Kurt Huber (Austria), Miroslav Solar (Czech Republic), Svend Eggert Jensen (Denmark), Basil Lewis (Israel), Bela Merkely (Hungary), Gilles Montalescot (France), Jaroslaw Trebacz (Poland), Pierluigi Tricoci (United States)

PERFUSE Study Group

C. Michael Gibson (Chair), Serge Korjian (Research Fellow), Yazan Daaboul (Research Fellow), Megan Yee (Biostatistician), Purva Jain (Biostatistician), Gerald Chi (Biostatistician), Donald Szlosek (Biostatistician), Rim Halaby (Research Fellow), Meghan Leitao (Administrative Director), Madeleine Cochet (Project Manager), Laura Goodell (Project Manager), Luke Rusowicz-Orazem (Project Manager), Nate Michalak (Project Manager)

CSL Behring, King of Prussia, Pennsylvania (Sponsor)

Denise D'Andrea (Senior Global Clinical Program Director, Cardiovascular and Metabolism), Lynn Nicolodi (Senior Director, Global Clinical Operations), Esther Lee (Clinical Program Manager), Gail Berman (Senior Clinical Program Director) Jenny Mears (Senior Clinical Scientist, Cardiovascular and Metabolism), Michael Giordani (Clinical Operations Lead, Cardiovascular), Mark Heise (Associate Director, Biostatistics), Lawrence Deckelbaum (Therapeutic Area Head, Cardiovascular and Metabolism), Jean Myers (Clinical Program Manager), Karen Goldstein (Unblinded Clinical Study Manager, NA), Lucinda Gilde (Senior Clinical Scientist, Cardiovascular and Metabolism), Steve Horchler (Data Management), Stefanie Auer (Regional Study Manager, EU), Ray Perone (Regional Study Manager, NA), Jacqui Cumming (Regional Study Manager, AUS), Michael Meisner (Unblinded Regional Study Manager, EU), Ortrud Harlinghaus (Regional Study Manager, EU), Shimin Wei (Senior Clinical Scientist), Elizabeth Bean (Manager, Clinical Supplies and Logistics), Joanna Grego (Pharmacovigilance), Charles Liss (Global Statistical Scientist), Michael Tortorici (Director, Clinical Pharmacology and Pharmacometrics), Samuel D. Wright (Pharmacology Lead), Andreas Gille (Translational Medicine Lead), Danielle Duffy (Clinical Program Director)

Independent Statistical Groups

PERFUSE Study Group, Quintiles

Data and Safety Monitoring Board

W. Douglas Weaver (Chair), Michel Bertrand, David Faxon, Bruce Molitoris, Jan Tijssen, David Waters, Irene Katzan (Non-Voting Member), Andrew Muir (Non-Voting Member), Gerry McConnel (Project Manager)

Clinical Events Committee

C5 Research (Ohio): Venu Menon (Chair), Vidyasagar Kalahasti, Jitendra Sharma, Nael Hawwa, Samuel Horr, Grant Reed, Brett Sperry, Newton Wiggins; Kim Brown (CEC Manager); Crissy Shaffer (CEC Manager)

Core Laboratories

Biomarker: Pacific Biomarkers (Seattle, Washington): Reynelle Warren; Specimen: Quintiles Laboratory: Gina Tai

North America Site Management and Monitoring

Duke Clinical Research Institute (North Carolina): John H. Alexander, Pierluigi Tricoci, Christopher Fordyce

Contract Research Organization

Quintiles

Appendix B - Enrolling Sites

Australia
National Lead Investigator: P. Aylward
18 patients enrolled, 5 sites: J. Amerena, J. Horowitz, W. Parsonage, W. van Gaal, M. Worthley

Austria
National Lead Investigator: K. Huber
7 patients enrolled, 3 sites: C. Brenner, K. Huber, I. Lang

Bulgaria
National Lead Investigator: N. Gotcheva
140 patients enrolled, 19 sites: B. Atzev, H. Benov, B. Dimov, V. Gelev, N. Gotcheva, E. Karadimova, R. Kasabov, Z. Koleva, M. Konteva, P. Lazov, A. Manolova, S. Marchev, D. Markov, N. Penkov, I. Petrov, D. Popov, D. Raev, G. Tonev, B. Zehirov

Canada
National Lead Investigator: P. Armstrong
25 patients enrolled, 9 sites: O. Bertrand, P. Cheung, D. Cleveland, A. Della Siega, S. Lavi, S. Mansour, B. Sussex, M. Vo, R. Welsh

Czech Republic
National Lead Investigator: M. Solar
104 patients enrolled, 16 sites: D. Alan, J. Brotanek, T. Budesinsky, L. Buncek, V. Dedek, L. Groch, M. Homza, T. Janota, P. Kala, Z. Klimsa, J. Maly, P. Reichert, S. Simek, J. Slaby, M. Solar, J. Vesely

Denmark
National Lead Investigator: S. Jensen
28 patients enrolled, 5 sites: A. Diederichsen, G. Gislason, S. Jensen, W. Nielsen, K. Thomsen

France
National Lead Investigator: G. Montalescot
3 patients enrolled, 5 sites: P. Coste, N. Delarche, B. Farah, G. Montalescot, V. Probst

Germany
National Lead Investigator: C. Bode
86 patients enrolled, 10 sites: H. Darius, H. Duengen, D. Duerschmied, H. Ince, C. Kadel, M. Karakas, B. Lemke, A. Schaefer, D. Schuster, U. Zeymer

Hungary
National Lead Investigator: B. Merkely
202 patients enrolled, 8 sites: B. Benczur, C. Dezsi, I. Horvath, R. Kiss, B. Merkely, P. Polgar, J. Tomcsanyi, I. Ungi

Israel
National Lead Investigator: B. Lewis
81 patients enrolled, 9 sites: R. Alcalai, S. Atar, R. Durst, D. Gavish, M. Halabi, T. Hayek, A. Katz, S. Kobal, M. Shechter

Italy
National Lead Investigator: G. Ambrosio
22 patients enrolled, 8 sites: G. Ambrosio, E. Corrada, M. D'Urbano, G. Morocutti, G. Piovaccari, M. Scherillo, B. Trimarco, M. Volpe

Netherlands
National Lead Investigator: J.H. Cornel
145 patients enrolled, 12 sites: R.W. Breedveld, J.H. Cornel, R.J. de Winter, F.R. Den Hartog, W. Hermans, J-P. Herrman, A.J.M. Oude Ophius, B. Rensing, P. Smits, H. Swart, R.P.T. Troquay, N. van Royen

Poland
National Lead Investigator: J. Trebacz
204 patients enrolled, 11 sites: M. Bronisz, W. Dubaniewicz, J. Kasprzak, G. Raczak, J. Szachniewicz, R. Szelemej, R. Targonski, J. Trebacz, D. Wojciechowski, B. Wozakowska-Kaplon, M. Zarebinski

Spain
National Lead Investigator: A. Betriu
17 patients enrolled, 12 sites: F. Cereto Castro, F. Fernandez Aviles, A. Fernandez Ortiz, A. Fillat, J.R. Gonzalez Juanatey, M. Jimenez Navarro, J. Merce Klein, L. Recasens, M. Sabate, J. Sanchis Fores, J.M. Vazquez Rodriguez, J.L. Zamorano

United Kingdom
National Lead Investigator: A. Gershlick
9 patients enrolled, 6 sites: A. Gershlick, M. Jain, A. Kabir, H. Kadr, J. Rocchiccioli, A. Zaman

United States
National Lead Investigator: P. Tricoci
167 patients enrolled, 46 sites: R. Andersen, D. Angiolilo, A. Bailey, H. Barrett, R. Black, E. Brilakis, J. Burchenal, A. Chu, R. Coram, M. Cuchel, J. Estess, W. French, G. Gershony, M. Ghali, P. Gurbel, J. Heitner, D. Hinchman, N. Jaffrani, T. Knickelbine, G. Koenig, M. Kontos, E. Kosinski, M. Kozak, P. Kumar, G. Larrain, D. Li, A. Meholick, V. Mehta, D. Miranda, R. Pradhan, A. Quyyumi, D. Roberts, J. Rossi, I. Sarembock, J. Saucedo, S. Sudarshan, J. Sweeny, P. Tricoci, A. Vasquez, J. Waltman, H. Wasserman, R. Weiss, J. Whitaker, L. White, M. Wilensky, A. Wiseman
